# Crystal structure of 6-hy­droxy-5-(2-meth­oxy­phenoxy)-2,2′-bipyrimidin-4(3*H*)-one

**DOI:** 10.1107/S2056989016009075

**Published:** 2016-06-17

**Authors:** Belakavadi K. Sagar, Hemmige S. Yathirajan, Jerry P. Jasinski, Christopher Glidewell

**Affiliations:** a Department of Studies in Chemistry, University of Mysore, Manasagangotri, Mysuru 570 006, India; b Department of Chemistry, Keene State College, 229 Main Street, Keene, NH 03435-2001, USA; c School of Chemistry, University of St Andrews, St Andrews, Fife KY16 9ST, UK

**Keywords:** crystal structure, supra­molecular structure, bi­pyrimidines, mol­ecular conformation, hydrogen bonding

## Abstract

In the title compound, C_15_H_12_N_4_O_4_, the dihedral angle between the heterocyclic rings is 12.60 (8)°, and that between the benzene ring and the adjacent heterocyclic ring is 85.14 (6)°. In the crystal, a combination of N—H⋯O and O—H⋯O hydrogen bonds link mol­ecules related by a glide plane into a *C*(5) C(6)[*R* ^2^_2_(9)] chain of rings, which is a distinctly different packing motif to those observed in hydrated modifications of this compound.

## Abstract 2

In the crystal, a combination of N—H⋯O and O—H⋯O hydrogen bonds links the mol­ecules of the title compounds into a chain of rings.

## Chemical context   

Pyrimidine derivatives exhibit a wide variety of biological actions (Önal & Yıldırım, 2007 ▸) and specific examples are of particular value in the treatment of cardiovascular diseases (Goldmann & Stoltefuss, 1991 ▸). One such derivative is bosentan, 4-*tert*-butyl-*N*-[6-(2-hy­droxy­eth­oxy)-5-(2-meth­oxyphen­oxy)-2-(pyrimidin-2-yl)pyrimidin-4-yl]benzene-1-sulfonamide, which is used in the treatment of pulmonary artery hypertension (Pearl *et al.*, 1999 ▸; Hoeper *et al.*, 2003 ▸; Kenyon & Nappi, 2003 ▸).
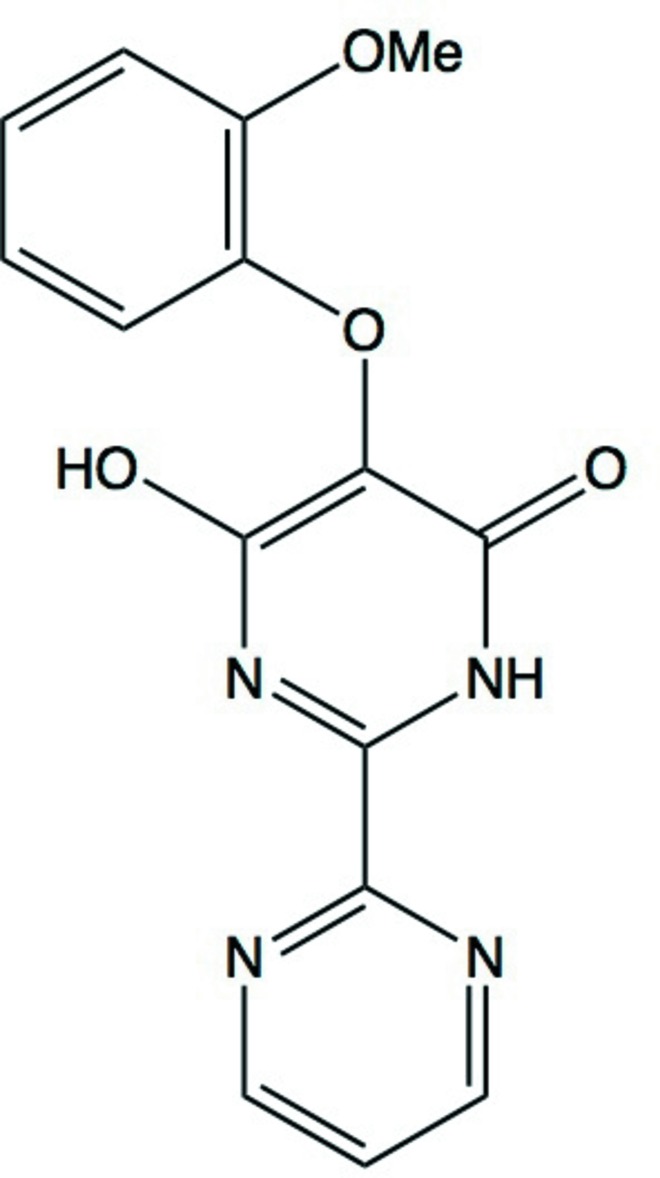


**Figure 1:**
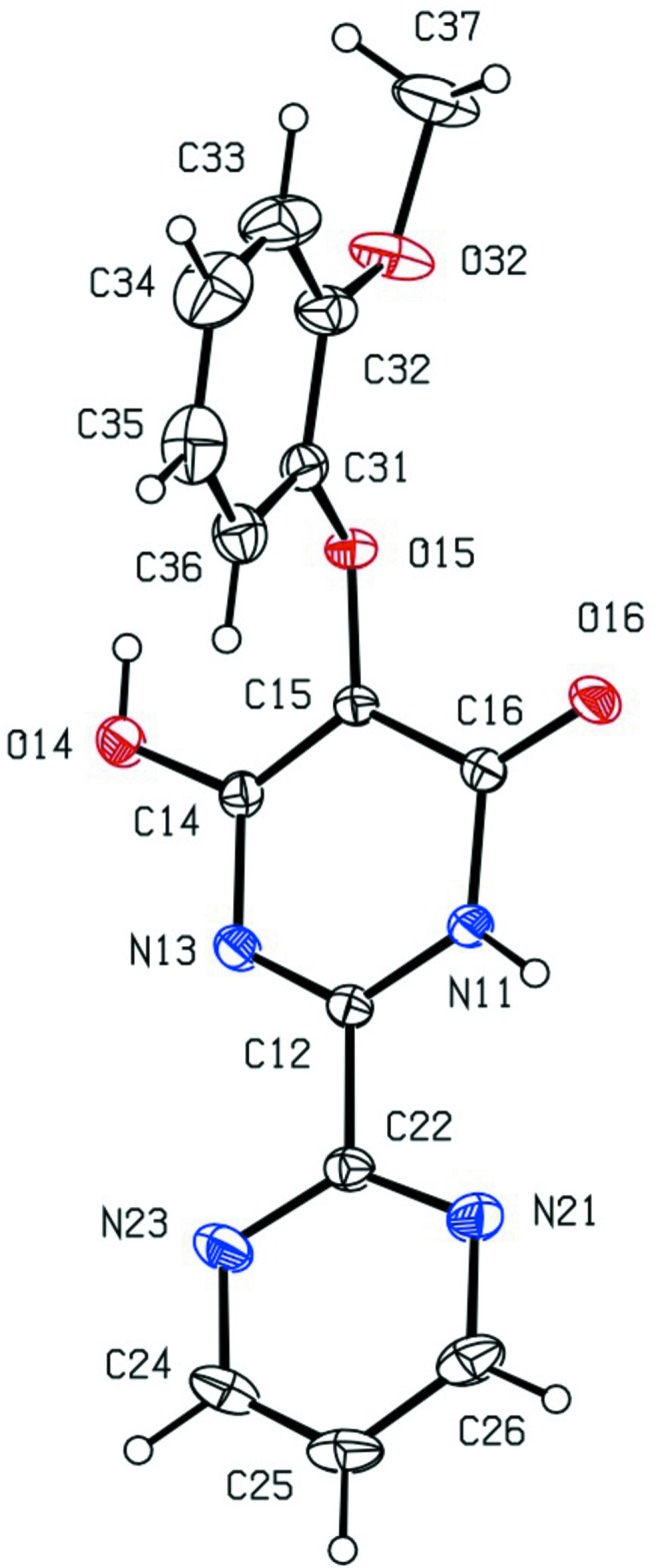
The mol­ecular structure of compound (I) showing displacement ellipsoids drawn at the 30% probability level.

4-Hy­droxy-5-(2-meth­oxy­phen­oxy)-2,2′-bipyrimidin-6(1*H*)-one (I) (Fig. 1 ▸) is an inter­mediate in the synthesis of bosentan (Rebelli *et al.*, 2013 ▸; Kompella *et al.*, 2014 ▸) and accordingly it is of inter­est to determine its crystal and mol­ecular structure, which we report here. Crystals of the anhydrous title compound (I) were obtained from a solution of a 1:1 mixture of di­methyl­sulfoxide and *N*,*N*-di­methyl­formamide in the presence of adipic acid: by contrast, a similar crystallization regime but omitting the adipic acid yielded the corresponding dihydrate (II) (Yamuna *et al.*, 2013 ▸), so permitting comparison of the anhydrous and hydrated forms.

## Structural commentary   

The bond distances in the ring containing atom N11 clearly show the presence of localized double bonds in the bonds C12=N13 and C14=C15 as well as the exocyclic C16=O16, fully consistent with the location of the H atoms on atoms N11 and O14, as deduced from difference maps and confirmed by the refinement. By contrast, the bond distances in the other heterocyclic ring indicate conventional aromatic-type delocalization.

At each of the sites C14, C31 and C32, the corresponding pairs of exocyclic O—C—N (at C14) or O—C—C angles (at C31 and C32) differ by almost 10°, as generally observed in the arenes of type ArO*R* when the substituent *R* lies close to the plane of the aryl ring (Seip & Seip, 1973 ▸; Ferguson *et al.*, 1996 ▸). Here atoms C15 and C37 (Fig. 1 ▸) are displaced from the plane of the aryl ring (C31–C36) by 0.219 (3) and 0.204 (4) Å, respectively, with both substituents displaced to the same side of the aryl ring. The C—O—C angles at atoms O15 and O32, 115.41 (12) and 117.65 (18)° respectively, and the C—O—H angle at atom O14 is 114.2 (16)°; are all significantly larger the the idealized tetra­hedral value of 109.5°.

The dihedral angle between the heterocyclic rings is 12.60 (8)° and that between the ring containing N11 and the aryl ring is 85.14 (6)°. Accordingly, the mol­ecule of (I) exhibits no inter­nal symmetry and thus the compound is conformationally chiral: the centrosymmetric space group confirms that (I) crystallizes as a conformational racemate.

## Supra­molecular inter­actions   

**Figure 2:**
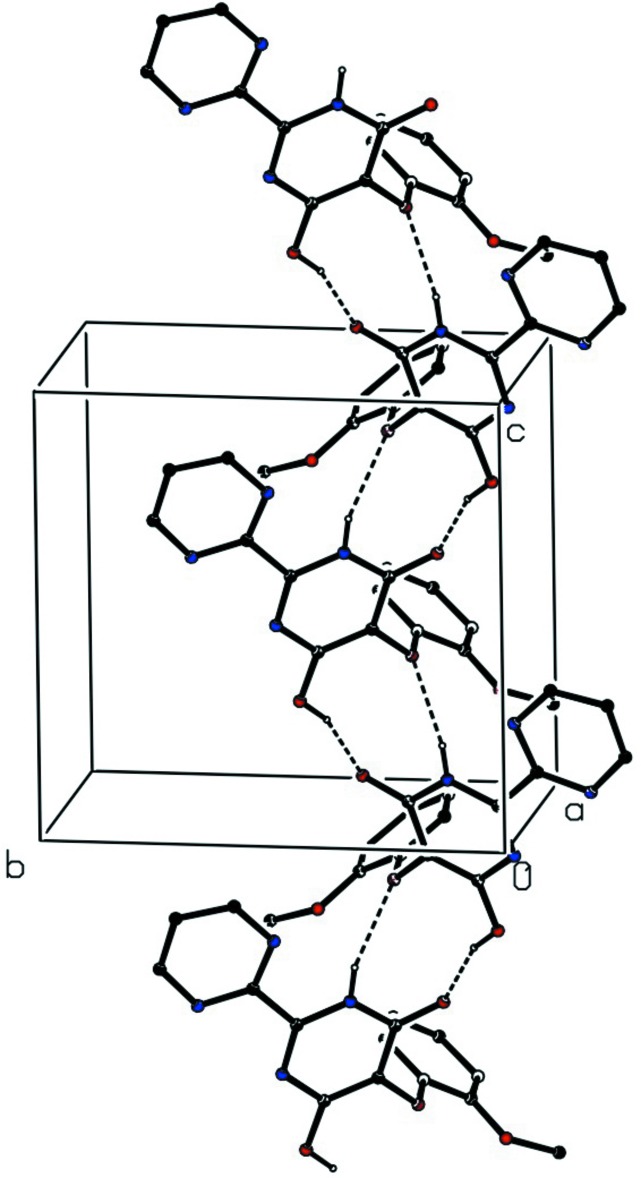
Part of the crystal structure of compound (I) showing the formation of a hydrogen-bonded *C*(5) *C*(6)[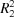)9)] chain of rings parallel to [001]. For the sake of clarity, the H atoms bonded to C atoms have all been omitted.

In the crystal, mol­ecules of (I) are linked by a combination of O—H⋯N and N—H⋯N hydrogen bonds (Table 1 ▸) to form a *C*(5) *C*(6)[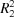
(9)] chain of rings running parallel to the [001] direction (Fig. 2 ▸): adjacent mol­ecules are related by glide-plane symmetry. Two chains of this type, related to one another by inversion, pass through each unit cell, but there are no direction-specific inter­actions between adjacent chains.

## Database survey   

In the dihydrate (II), an extensive series of hydrogen bonds, encompassing N—H⋯O, O—H⋯N and O—H⋯O types links the mol­ecular components into a complex sheet structure (Yamuna *et al.*, 2013 ▸), in contrast to the rather simple chains in (I) reported here. A sheet structure, built from a combination of the same three types of hydrogen bond is found also in the structure of bosentan monohydrate (Kaur *et al.*, 2013 ▸).

## Synthesis and crystallization   

A sample of compound (I) was a gift from Cadila Pharmaceuticals Ltd, Ahmedabad, Gujarat, India. Colourless plates of the anhydrous compound (I) were grown by slow evaporation, at room temperature of a solution of (I) in a mixture of di­methyl­sulfoxide and *N*,*N*-di­methyl­formamide (1:1, *v*/*v*) containing an excess of adipic acid (hexane-1,6-dioic acid).

## Refinement   

Crystal data, data collection and structure refinement details are summarized in Table 2 ▸. All H atoms were located in difference maps. The H atoms bonded to C atoms were then treated as riding atoms in geometrically idealized positions with C—H distances 0.93 Å (aromatic and heteroaromatic) or 0.96 Å (CH_3_) and with *U*_iso_(H) = *kU*_eq_(C) where *k* = 1.5 for the methyl group, which was permitted to rotate but not to tilt and 1.2 for all other H atoms bonded to C atoms. For the H atoms bonded to O or N atoms, the atomic coordinates were refined with *U*_iso_(H) = 1.5*U*_eq_(O) or 1.2*U*_eq_(N), giving the O—H and N—H distances shown in Table 1 ▸.

## Supplementary Material

Crystal structure: contains datablock(s) global, I. DOI: 10.1107/S2056989016009075/hb7590sup1.cif

Structure factors: contains datablock(s) I. DOI: 10.1107/S2056989016009075/hb7590Isup2.hkl

Supporting information file. DOI: 10.1107/S2056989016009075/hb7590Isup3.cml

CCDC reference: 1483503

Additional supporting information: crystallographic information; 3D view; checkCIF report

## Figures and Tables

**Table 1:** Hydrogen-bond geometry (Å, °)

*D*—H⋯*A*	*D*—H	H⋯*A*	*D*⋯*A*	*D*—H⋯*A*
N11—H11⋯O15^i^	0.855 (19)	2.257 (19)	2.9733 (18)	141.4 (17)
O14—H14⋯O16^ii^	0.85 (2)	1.80 (2)	2.6117 (18)	160 (2)

Symmetry codes: (i) 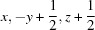; (ii) 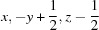.

**Table 2:** Experimental details

Crystal data	
Chemical formula	C_15_H_12_N_4_O_4_
*M*_r_	312.29
Crystal system, space group	Monoclinic, *P*2_1_/*c*
Temperature (K)	298
*a*, *b*, *c* (Å)	12.1863 (9), 10.7079 (8), 11.1726 (8)
β (°)	105.412 (8)
*V* (Å^3^)	1405.48 (19)
*Z*	4
Radiation type	Mo *K*α
μ (mm^−1^)	0.11
Crystal size (mm)	0.49 × 0.46 × 0.28

Data collection	
Diffractometer	Agilent Xcalibur, Eos, Gemini CCD
Absorption correction	Multi-scan (*SADABS*; Sheldrick, 2003 ▸)
*T*_min_, *T*_max_	0.812, 0.969
No. of measured, independent and observed [*I* > 2σ(*I*)] reflections	7121, 3113, 2311
*R*_int_	0.037
(sin θ/λ)_max_ (Å^−1^)	0.650

Refinement	
*R*[*F* ^2^ > 2σ(*F* ^2^)], *wR*(*F* ^2^), *S*	0.051, 0.134, 1.07
No. of reflections	3113
No. of parameters	215
H-atom treatment	H atoms treated by a mixture of independent and constrained refinement
Δρ_max_, Δρ_min_ (e Å^−3^)	0.26, −0.25

Computer programs: *CrysAlis PRO* (Agilent, 2014 ▸), *SHELXS97* (Sheldrick, 2008 ▸), *SHELXL2014* (Sheldrick, 2015 ▸) and *PLATON* (Spek, 2009 ▸).

## supplementary crystallographic information

### Crystal data

**Table:**

C_15_H_12_N_4_O_4_	*F*(000) = 648
*M**_r_* = 312.29	*D*_x_ = 1.476 Mg m^−^^3^
Monoclinic, *P*2_1_/*c*	Mo *K*α radiation, λ = 0.71073 Å
*a* = 12.1863 (9) Å	Cell parameters from 3266 reflections
*b* = 10.7079 (8) Å	θ = 3.5–29.2°
*c* = 11.1726 (8) Å	µ = 0.11 mm^−^^1^
β = 105.412 (8)°	*T* = 298 K
*V* = 1405.48 (19) Å^3^	Plate, colourles
*Z* = 4	0.49 × 0.46 × 0.28 mm

### Data collection

**Table:**

Agilent Xcalibur, Eos, Gemini CCD diffractometer	2311 reflections with *I* > 2σ(*I*)
Detector resolution: 16.0416 pixels mm^-1^	*R*_int_ = 0.037
φ and ω scans	θ_max_ = 27.5°, θ_min_ = 3.5°
Absorption correction: multi-scan (SADABS; Sheldrick, 2003)	*h* = −15→15
*T*_min_ = 0.812, *T*_max_ = 0.969	*k* = −13→10
7121 measured reflections	*l* = −14→14
3113 independent reflections	

### Refinement

**Table:**

Refinement on *F*^2^	Primary atom site location: structure-invariant direct methods
Least-squares matrix: full	Secondary atom site location: difference Fourier map
*R*[*F*^2^ > 2σ(*F*^2^)] = 0.051	Hydrogen site location: mixed
*wR*(*F*^2^) = 0.134	H atoms treated by a mixture of independent and constrained refinement
*S* = 1.07	*w* = 1/[σ^2^(*F*_o_^2^) + (0.0582*P*)^2^ + 0.1493*P*] where *P* = (*F*_o_^2^ + 2*F*_c_^2^)/3
3113 reflections	(Δ/σ)_max_ < 0.001
215 parameters	Δρ_max_ = 0.26 e Å^−^^3^
0 restraints	Δρ_min_ = −0.25 e Å^−^^3^

### Special details

**Table:**

Geometry. All esds (except the esd in the dihedral angle between two l.s. planes) are estimated using the full covariance matrix. The cell esds are taken into account individually in the estimation of esds in distances, angles and torsion angles; correlations between esds in cell parameters are only used when they are defined by crystal symmetry. An approximate (isotropic) treatment of cell esds is used for estimating esds involving l.s. planes.

### Fractional atomic coordinates and isotropic or equivalent isotropic displacement parameters (Å^2^)

**Table:**

	*x*	*y*	*z*	*U*_iso_*/*U*_eq_	
N11	0.18153 (12)	0.35790 (14)	0.63063 (13)	0.0295 (3)	
H11	0.1817 (15)	0.3477 (17)	0.7066 (18)	0.035*	
C12	0.14803 (12)	0.47018 (16)	0.58084 (14)	0.0257 (4)	
N13	0.15092 (11)	0.50269 (13)	0.46986 (12)	0.0288 (3)	
C14	0.19721 (13)	0.41910 (16)	0.40542 (14)	0.0259 (4)	
C15	0.23862 (13)	0.30650 (15)	0.45349 (14)	0.0246 (4)	
C16	0.22954 (14)	0.26765 (16)	0.57208 (15)	0.0286 (4)	
O14	0.20095 (11)	0.45956 (12)	0.29445 (11)	0.0384 (3)	
H14	0.2235 (18)	0.405 (2)	0.251 (2)	0.058*	
O15	0.29101 (9)	0.22750 (11)	0.38791 (10)	0.0286 (3)	
O16	0.26156 (12)	0.16663 (12)	0.62240 (11)	0.0435 (4)	
N21	0.10457 (13)	0.51230 (15)	0.77235 (13)	0.0382 (4)	
C22	0.11108 (13)	0.55897 (16)	0.66440 (15)	0.0287 (4)	
N23	0.09267 (14)	0.67630 (15)	0.62621 (15)	0.0442 (4)	
C24	0.06898 (19)	0.7543 (2)	0.7096 (2)	0.0534 (6)	
H24	0.0564	0.8380	0.6881	0.064*	
C25	0.06238 (17)	0.7179 (2)	0.8239 (2)	0.0503 (6)	
H25	0.0467	0.7743	0.8805	0.060*	
C26	0.07998 (16)	0.5938 (2)	0.85148 (18)	0.0472 (5)	
H26	0.0746	0.5653	0.9283	0.057*	
C31	0.41008 (14)	0.22844 (17)	0.42471 (15)	0.0305 (4)	
C32	0.46353 (16)	0.1335 (2)	0.37687 (17)	0.0417 (5)	
C33	0.58106 (19)	0.1307 (3)	0.4082 (2)	0.0639 (7)	
H33	0.6184	0.0678	0.3771	0.077*	
C34	0.64289 (19)	0.2205 (3)	0.4850 (3)	0.0713 (8)	
H34	0.7220	0.2185	0.5043	0.086*	
C35	0.59019 (18)	0.3127 (3)	0.5336 (2)	0.0595 (6)	
H35	0.6330	0.3720	0.5868	0.071*	
C36	0.47253 (15)	0.3169 (2)	0.50285 (17)	0.0414 (5)	
H36	0.4358	0.3795	0.5351	0.050*	
O32	0.39382 (13)	0.05064 (15)	0.30120 (14)	0.0585 (5)	
C37	0.4443 (3)	−0.0572 (3)	0.2648 (2)	0.0755 (8)	
H37A	0.4889	−0.0336	0.2095	0.113*	
H37B	0.3857	−0.1143	0.2233	0.113*	
H37C	0.4923	−0.0968	0.3369	0.113*	

### Atomic displacement parameters (Å^2^)

**Table:**

	*U*^11^	*U*^22^	*U*^33^	*U*^12^	*U*^13^	*U*^23^
N11	0.0418 (8)	0.0269 (8)	0.0233 (7)	0.0053 (6)	0.0148 (6)	0.0020 (6)
C12	0.0255 (8)	0.0249 (9)	0.0261 (8)	0.0005 (7)	0.0059 (6)	−0.0018 (7)
N13	0.0345 (7)	0.0239 (8)	0.0278 (7)	0.0035 (6)	0.0078 (6)	0.0006 (6)
C14	0.0298 (8)	0.0261 (9)	0.0216 (7)	−0.0028 (7)	0.0063 (6)	−0.0010 (7)
C15	0.0283 (8)	0.0233 (9)	0.0235 (7)	0.0008 (7)	0.0090 (6)	−0.0023 (7)
C16	0.0342 (9)	0.0251 (10)	0.0283 (8)	0.0022 (7)	0.0112 (6)	0.0005 (7)
O14	0.0632 (8)	0.0300 (8)	0.0255 (6)	0.0066 (6)	0.0178 (6)	0.0038 (5)
O15	0.0310 (6)	0.0293 (7)	0.0267 (6)	0.0032 (5)	0.0098 (4)	−0.0058 (5)
O16	0.0722 (9)	0.0286 (8)	0.0360 (7)	0.0176 (7)	0.0256 (6)	0.0095 (6)
N21	0.0455 (9)	0.0392 (10)	0.0327 (8)	−0.0007 (7)	0.0155 (6)	−0.0068 (7)
C22	0.0273 (8)	0.0283 (10)	0.0304 (8)	−0.0007 (7)	0.0074 (6)	−0.0054 (7)
N23	0.0592 (10)	0.0287 (9)	0.0473 (9)	0.0076 (8)	0.0189 (8)	−0.0052 (7)
C24	0.0661 (14)	0.0319 (12)	0.0638 (14)	0.0109 (10)	0.0198 (11)	−0.0135 (10)
C25	0.0466 (11)	0.0519 (14)	0.0544 (13)	0.0037 (10)	0.0169 (9)	−0.0267 (11)
C26	0.0511 (11)	0.0596 (15)	0.0354 (10)	−0.0023 (11)	0.0194 (9)	−0.0162 (10)
C31	0.0315 (8)	0.0330 (10)	0.0278 (8)	0.0055 (7)	0.0091 (6)	0.0059 (7)
C32	0.0456 (11)	0.0453 (13)	0.0333 (9)	0.0162 (9)	0.0092 (8)	0.0034 (9)
C33	0.0484 (13)	0.085 (2)	0.0592 (14)	0.0317 (13)	0.0169 (11)	0.0048 (14)
C34	0.0340 (12)	0.101 (2)	0.0756 (17)	0.0110 (13)	0.0089 (11)	0.0157 (17)
C35	0.0424 (12)	0.0687 (17)	0.0600 (14)	−0.0114 (11)	0.0008 (10)	0.0076 (13)
C36	0.0394 (10)	0.0399 (12)	0.0438 (11)	−0.0031 (9)	0.0089 (8)	0.0016 (9)
O32	0.0649 (9)	0.0506 (10)	0.0543 (9)	0.0259 (8)	0.0057 (7)	−0.0188 (8)
C37	0.113 (2)	0.0547 (16)	0.0594 (15)	0.0433 (15)	0.0238 (14)	−0.0059 (13)

### Geometric parameters (Å, º)

**Table:**

N11—C12	1.342 (2)	C26—N21	1.332 (2)
N11—H11	0.86 (2)	C16—O16	1.234 (2)
C12—N13	1.297 (2)	C26—H26	0.9300
C12—C22	1.484 (2)	C31—C36	1.373 (3)
N13—C14	1.361 (2)	C31—C32	1.389 (3)
C14—C15	1.361 (2)	C32—O32	1.357 (2)
C15—C16	1.421 (2)	C32—C33	1.381 (3)
C16—N11	1.381 (2)	C33—C34	1.373 (4)
C14—O14	1.3255 (19)	C33—H33	0.9300
C15—O15	1.3816 (18)	C34—C35	1.367 (4)
O14—H14	0.85 (2)	C34—H34	0.9300
O15—C31	1.3990 (19)	C35—C36	1.384 (3)
N21—C22	1.327 (2)	C35—H35	0.9300
C22—N23	1.327 (2)	C36—H36	0.9300
N23—C24	1.339 (2)	O32—C37	1.418 (3)
C24—C25	1.358 (3)	C37—H37A	0.9600
C24—H24	0.9300	C37—H37B	0.9600
C25—C26	1.368 (3)	C37—H37C	0.9600
C25—H25	0.9300		
			
C12—N11—C16	123.44 (14)	N21—C26—H26	118.8
C12—N11—H11	116.5 (13)	C25—C26—H26	118.8
C16—N11—H11	119.4 (13)	C36—C31—C32	120.81 (17)
N13—C12—N11	123.65 (15)	O15—C31—C32	115.95 (15)
N13—C12—C22	121.28 (15)	O15—C31—C36	123.24 (16)
N11—C12—C22	115.02 (14)	O32—C32—C31	116.00 (16)
C12—N13—C14	116.70 (14)	O32—C32—C33	125.34 (19)
O14—C14—N13	113.69 (14)	C15—O15—C31	115.41 (12)
O14—C14—C15	123.87 (15)	C32—O32—C37	117.65 (18)
C15—C14—N13	122.41 (14)	C33—C32—C31	118.7 (2)
C14—C15—O15	120.54 (13)	C34—C33—C32	120.2 (2)
C14—C15—C16	120.98 (15)	C34—C33—H33	119.9
O15—C15—C16	118.47 (14)	C32—C33—H33	119.9
O16—C16—N11	121.35 (15)	C35—C34—C33	121.1 (2)
O16—C16—C15	126.03 (16)	C35—C34—H34	119.4
N11—C16—C15	112.61 (14)	C33—C34—H34	119.4
C14—O14—H14	114.2 (16)	C34—C35—C36	119.4 (2)
C22—N21—C26	115.78 (17)	C34—C35—H35	120.3
N23—C22—N21	127.09 (16)	C36—C35—H35	120.3
N23—C22—C12	117.26 (15)	C31—C36—C35	119.9 (2)
N21—C22—C12	115.59 (15)	C31—C36—H36	120.1
C22—N23—C24	114.59 (17)	C35—C36—H36	120.1
N23—C24—C25	123.6 (2)	O32—C37—H37A	109.5
N23—C24—H24	118.2	O32—C37—H37B	109.5
C25—C24—H24	118.2	H37A—C37—H37B	109.5
C24—C25—C26	116.47 (19)	O32—C37—H37C	109.5
C24—C25—H25	121.8	H37A—C37—H37C	109.5
C26—C25—H25	121.8	H37B—C37—H37C	109.5
N21—C26—C25	122.44 (19)		
			
C16—N11—C12—N13	4.3 (2)	N11—C12—C22—N21	−6.7 (2)
C16—N11—C12—C22	−173.20 (14)	N21—C22—N23—C24	2.4 (3)
N11—C12—N13—C14	−4.0 (2)	C12—C22—N23—C24	−174.60 (16)
C22—C12—N13—C14	173.33 (13)	C22—N23—C24—C25	−0.9 (3)
C12—N13—C14—O14	−177.94 (14)	N23—C24—C25—C26	−0.8 (3)
C12—N13—C14—C15	0.1 (2)	C22—N21—C26—C25	0.0 (3)
O14—C14—C15—O15	1.7 (2)	C24—C25—C26—N21	1.3 (3)
N13—C14—C15—O15	−176.13 (13)	C15—O15—C31—C36	−12.1 (2)
O14—C14—C15—C16	−178.56 (15)	C15—O15—C31—C32	168.10 (14)
N13—C14—C15—C16	3.6 (2)	C36—C31—C32—O32	179.61 (17)
C12—N11—C16—O16	178.32 (16)	O15—C31—C32—O32	−0.6 (2)
C12—N11—C16—C15	−0.4 (2)	C36—C31—C32—C33	−0.8 (3)
C14—C15—C16—O16	178.06 (17)	O15—C31—C32—C33	178.93 (17)
O15—C15—C16—O16	−2.2 (3)	O32—C32—C33—C34	179.5 (2)
C14—C15—C16—N11	−3.2 (2)	C31—C32—C33—C34	−0.1 (3)
O15—C15—C16—N11	176.48 (13)	C32—C33—C34—C35	1.1 (4)
C14—C15—O15—C31	100.24 (17)	C33—C34—C35—C36	−1.2 (4)
C16—C15—O15—C31	−79.49 (18)	C32—C31—C36—C35	0.7 (3)
C26—N21—C22—N23	−2.0 (3)	O15—C31—C36—C35	−179.05 (17)
C26—N21—C22—C12	175.03 (15)	C34—C35—C36—C31	0.3 (3)
N13—C12—C22—N23	−6.9 (2)	C33—C32—O32—C37	9.3 (3)
N11—C12—C22—N23	170.67 (15)	C31—C32—O32—C37	−171.21 (19)
N13—C12—C22—N21	175.73 (14)		

### Hydrogen-bond geometry (Å, º)

**Table:**

*D*—H···*A*	*D*—H	H···*A*	*D*···*A*	*D*—H···*A*
N11—H11···O15^i^	0.855 (19)	2.257 (19)	2.9733 (18)	141.4 (17)
O14—H14···O16^ii^	0.85 (2)	1.80 (2)	2.6117 (18)	160 (2)

Symmetry codes: (i) *x*, −*y*+1/2, *z*+1/2; (ii) *x*, −*y*+1/2, *z*−1/2.

## References

[bb1] Agilent (2014). *CrysAlis PRO*. Agilent Technologies Ltd, Yarnton, England.

[bb2] Ferguson, G., Glidewell, C. & Patterson, I. L. J. (1996). *Acta Cryst.* C**52**, 420–423.

[bb3] Goldmann, S. & Stoltefuss, J. (1991). *Angew. Chem. Int. Ed. Engl.* **30**, 1559–1578.

[bb4] Hoeper, M., Taha, N., Bekjarova, A., Gatzke, R. & Spiekerkoetter, E. (2003). *Eur. Respir. J.* **22**, 330–334. 10.1183/09031936.03.00008003 12952269

[bb5] Kaur, M., Jasinski, J. P., Keeley, A. C., Yathirajan, H. S., Betz, R., Gerber, T. & Butcher, R. J. (2013). *Acta Cryst.* E**69**, o12–o13. 10.1107/S1600536812048969 PMC3588285 23476382

[bb6] Kenyon, K. W. & Nappi, J. M. (2003). *Ann. Pharmacother.* **37**, 1055–1062. 10.1345/aph.1C256 12841819

[bb7] Kompella, A., Kasa, S., Balina, V. S., Kusumba, S., Adibhatla, B. R. K. & Muddasani, P. R. (2014). *Sci. J. Chem.* **2**, 9–15.

[bb8] Önal, Z. & Yıldırım, İ. (2007). *Heterocycl. Commun.* **13**, 113–120.

[bb9] Pearl, J. M., Wellmann, S. A., McNamara, J. L., Lombardi, J. P., Wagner, C. J., Raake, J. L. & Nelson, D. P. (1999). *Ann. Thorac. Surg.* **68**, 1714–21 discussion 1721–1714-21; discussion 1722. 10.1016/s0003-4975(99)00988-1 10585047

[bb10] Rebelli, P., Yerrabelly, J. R., Yalamanchili, B. K., Kommera, R., Ghojala, V. R. & Bairy, K. R. (2013). *Org. Process Res. Dev.* **17**, 1021–1026.

[bb11] Seip, H. M. & Seip, R. (1973). *Acta Chem. Scand.* **27**, 4024–4027.

[bb12] Sheldrick, G. M. (2003). *SADABS*. University of Göttingen, Germany.

[bb13] Sheldrick, G. M. (2008). *Acta Cryst.* A**64**, 112–122. 10.1107/S0108767307043930 18156677

[bb14] Sheldrick, G. M. (2015). *Acta Cryst.* C**71**, 3–8.

[bb15] Spek, A. L. (2009). *Acta Cryst.* D**65**, 148–155. 10.1107/S090744490804362X PMC2631630 19171970

[bb16] Yamuna, T. S., Jasinski, J. P., Anderson, B. J., Yathirajan, H. S. & Kaur, M. (2013). *Acta Cryst.* E**69**, o1707–o1708. 10.1107/S1600536813028900 PMC3884357 24454133

